# Interference Mitigation Schemes for Wireless Body Area Sensor Networks: A Comparative Survey

**DOI:** 10.3390/s150613805

**Published:** 2015-06-11

**Authors:** Thien T.T. Le, Sangman Moh

**Affiliations:** Department of Computer Engineering, Chosun University, 309 Pilmun-daero, Dong-gu, Gwangju 501-759, Korea; E-Mail: thanhthien92003@yahoo.com

**Keywords:** wireless body area sensor network, interference mitigation, coexistence, power control, medium access control, cognitive radio

## Abstract

A wireless body area sensor network (WBASN) consists of a coordinator and multiple sensors to monitor the biological signals and functions of the human body. This exciting area has motivated new research and standardization processes, especially in the area of WBASN performance and reliability. In scenarios of mobility or overlapped WBASNs, system performance will be significantly degraded because of unstable signal integrity. Hence, it is necessary to consider interference mitigation in the design. This survey presents a comparative review of interference mitigation schemes in WBASNs. Further, we show that current solutions are limited in reaching satisfactory performance, and thus, more advanced solutions should be developed in the future.

## 1. Introduction

A wireless body area sensor network (WBASN) is a wireless sensor network composed of short range, low power, and variable-data-rate sensors placed within, on, or around a human body to monitor biological signals and other functions such as movement patterns [1]. A WBASN system consists of biomedical sensor nodes for monitoring physiological data such as temperature, blood pressure, electrocardiography (ECG), electroencephalography (EEG), electromyography, and heart rate. These sensors may be wearable or implanted devices that collect and transmit vital signals to the coordinator. The collected data can then be forwarded to a hospital for medical purposes.

The applications of WBASNs can be categorized into two main fields: (1) medical applications that include remote patient monitoring, rehabilitation, biofeedback, and assisted living and (2) nonmedical applications that include fitness, performance and wellness monitoring, biometrics, and notification management [2]. Of course, for the medical and healthcare applications of WBASNs, the quality and continuity of signals are extremely important. However, radio propagation in WBASNs is dynamic due to the mobility of a human body, and different human tissues may be affected differently by biosensor signals. It is clearly shown in [3] that the quality of signals is affected by the propagation around the human body in accordance with channel models. Also, the effect of different kinds of human tissues to lossy signals is shown in the frequency range between 10 kHz and 1 GHz.

In [4], the requirements of WBASNs based on the IEEE 802.15.6 standard are summarized as follows: the bit rate of a link is in the range of 10 kbps to 10 Mbps, packet error rate should be less than 10% for a 256 octet payload for 95% of links, and the time to join or leave a network is less than 3 s. The communication architecture of WBASNs is divided into three tiers: intra-WBASN communication (tier 1), inter-WBASN communication (tier 2), and beyond-WBASN communication (tier 3).

Two standards, IEEE 802.15.4 [5] and IEEE 802.15.6 [1], cover WBASN technology. The IEEE 802.15.4 standard defines the physical (PHY) and medium access control (MAC) specification for low-rate wireless personal area networks at short range (up to 100 m) [5]. The IEEE 802.15.6 standard defines the PHY and MAC layers for WBASNs in short-range wireless communication within, on, or around the human body [1]. More specifically, the IEEE 802.15.4 standard is implemented into a specific application of IEEE 24451 standard [6]. The real application is a neurorecording system that aims to collect data of patients. The system consists of electrodes and a wireless module. The electrodes collect EEG signals of human head, and the wireless module uses different communication standards. In this application, three communication standards of IEEE 802.11, IEEE 802.15.1 and IEEE 802.15.4 are used. Furthermore, the MAC protocols of the two standards are compared in [7], where it is concluded that IEEE 802.15.6 is superior to IEEE 802.15.4 in terms of packet loss ratio, average delay, and network throughput. It is also shown that IEEE 802.15.6 carrier sense multiple access with collision avoidance (CSMA/CA) consumes more energy than IEEE 802.15.4 CSMA/CA. In [7], the design challenges of WBASNs are addressed, focusing on the MAC protocol, radio channel, and power consumption.

A WBASN is set up for an individual human body, and therefore, in many practical environments multiple WBASNs are unavoidable. Moreover, the human body is mobile, and the sensor devices associated with arms, legs, *et al*., on a human body are also mobile within a limited range. In such operation conditions, intra-WBASN and inter-WBASN interference may severely impair transmission. Therefore, in scenarios involving mobility or overlapped WBASNs, unstable signal integrity may spatially and temporally degrade network performance. For example, in a hospital environment, where many WBASNs are used for medical purposes, nearby WBASNs or other equipment operating at the same frequency band cause interference, and the lost signals may lead to a dangerous situation in the medical systems. Hence, it is considerably necessary to consider interference mitigation in the design.

WBASN interference can be categorized into three types: (1) Intra-WBASN interference occurs among sensor nodes within the same WBASN; (2) inter-WBASN interference occurs among WBASNs working at the same frequency band; and (3) inter-domain interference or cross-interference occurs between a WBASN and other wireless networks (e.g., Bluetooth, Zigbee, or WiFi) [7]. These forms of interference should be avoided or mitigated to ensure signal quality in WBASNs.

In this paper, existing interference mitigation schemes for WBASNs are extensively surveyed and discussed. The interference mitigation schemes are categorized into power control approach, MAC approach, cognitive radio approach, ultra wideband (UWB) approach, and signal processing approach. The important aspects of coexistence and interference in WBASNs are investigated first. Various interference mitigation schemes are then reviewed and compared. Some open issues and challenges are also discussed.

This paper is organized as follows: In the following section, the key points on coexistence and interference in WBASNs are introduced. The interference mitigation schemes are reviewed in detail in Section 3, and they are qualitatively compared and discussed in Section 4. In Section 5, open issues and challenges are discussed. Finally, the paper is concluded in Section 6.

## 2. Coexistence and Interference in WBASNs

**Figure 1:**
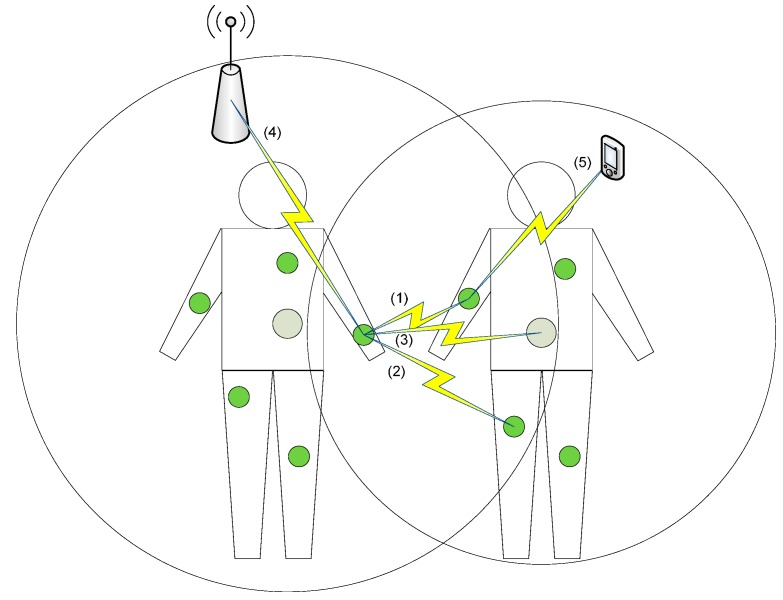
Interference in WBASNs.

This section introduces coexistence interference in WBASNs. Inter-WBASN coexistence and interference are reviewed first, and then inter-domain coexistence and interference are summarized. In Figure 1, the inter-WBASN interference can occur in wireless links (1), (2), and (3), whereas inter-domain interference can occur in wireless links (4) and (5).

### 2.1. Inter-WBASN Coexistence and Interference

In [8], experimentation is used to investigate the coexistence of WBASNs with regard to the inter-user interference effect. Degradation of performance in terms of packet delivery ratio and packet rate is clearly taken into account while varying the number of WBASNs and their transmission data rate. In the experiment, eight WBASNs were set up in which one consisted of a gateway and several nodes operating in the 2.4 GHz band. During the experiment, each WBASN sequentially switched on and transmitted until all WBASNs were transmitting simultaneously. The data rate of each WBASN was increased each minute until the maximum value was reached. The results of this experiment show that increasing the number of WBASNs adversely affects their packet delivery ratio (PDR) and packet rate. More specifically, at the maximum transmission rate, a large number of WBASNs joined to the network causes high degradation of PDR. When all WBASNs transmit simultaneously, increasing the data rate at each WBASN reduces the PDR. It is clear that an increase in the number of WBASNs and their transmission data rate will cause high performance degradation in WBASNs.

Co-channel interference among nonoverlapping WBASNs is analyzed in [9]. The network topology was modeled in a two-dimensional geometric representation in which each WBASN transmission range was modeled as a unit disk graph. Further, the network topology includes one tagged WBASN and interfering WBASNs and was developed into an advanced geometrical probability function. Based on that model, a gamma distribution function was used to approximate the total interference in the WBASN. The impact of interference was analyzed in terms of the boundary network distance to preserve the acceptable SINR (signal to interference plus noise ratio). For example, the minimum network distance should be larger than 7 to 12.5 m to guarantee the SINR of the boundary nodes and at least 9 to 18 m to ensure the average SINR requirement of the WBASN.

However, it is necessary to investigate the performance of the multiple access schemes for coexistence of WBASNs. In [10], the performance of three classic multiple access schemes is compared with respect to the probability of collision, the statistics of SINR, and bit error rate (BER). More specifically, the authors conducted the mathematical analysis to focus on: (1) the probability that one piconet collides with other piconets; and (2) the theoretical mean SINR for code division multiple access (CDMA), time division multiple access (TDMA), and frequency division multiple access (FDMA) schemes. The simulation results based on simultaneous measurement of signal and interference clearly show that TDMA is the best option in terms of BER and SINR in co-channel interference mitigation, and CDMA is not suitable. Otherwise, FDMA is the best solution for interference mitigation in uncoordinated WBASN networks.

In [11], dynamic coexistence of IEEE 802.15.4-based WBASNs is investigated by using the theoretical analysis of successful transmission. The author analyzed the probability of successful beacon and data transmission of a WBASN in a dynamic hospital environment. The simulation shows that the number of lost beacons increases when the number of coexisting WBASNs changes. In the case of high-data-rate transmission, data loss is higher in the presence of interference.

A mathematical model of interference among WBASNs in ISM (industrial, scientific, and medical) band is introduced in [12]. An experiment was set up in hospital, office, and home environments in Italy to measure the performance of WBASNs. The probability distribution function (PDF) of interference is defined based on those measurements. The maximum likelihood criteria are used to describe the PDF of the interference power.

In [13], intra-WBASN and inter-WBASN interference is analyzed by considering the cognitive radio system-based WBASN (CR-WBASN). The CR-WBASN uses a simplified human body model consisting of six cylinders. The frequency band is from 900 to 9000 MHz and is used by a UWB antenna. The simulation results show that the simplified model can be used to investigate and test the CR-WBASN.

The most important task in a dynamic multiple-WBASN environment is to detect and predict the coexistence of WBASNs. As a learning-based method, the naive-Bayesian-supervised learning method [14] was applied to detect whether a WBASN moves or not. Coexistence can be categorized into four states: static, semi-dynamic, dynamic, and none (no interference). The average packet reception ratio (PRR) and SINR are used as variables in the learning-based method to detect interference by comparing the thresholds of PRR and SINR. Four types of interference cases were also considered in the experiment to generate the training data set. In a multiple-WBASN environment, a WBASN consists of one coordinator node and four sensor nodes.

Interference occurs in a WBASN working in the ISM band. In addition, IEEE 802.15.6 UWB-based WBASNs can receive interference from other UWB devices. In [15], another interference study analyzes UWB-based WBASNs. The interference characterization is discussed with regard to the SINR, emitting power, and temporal model. The simulation results show that the BER performance of the UWB-based WBASNs depends on the power signal of the interferers working on IEEE 802.15.4a (piconets) or IEEE 802.15.4f (RFID) systems.

**Figure 2:**
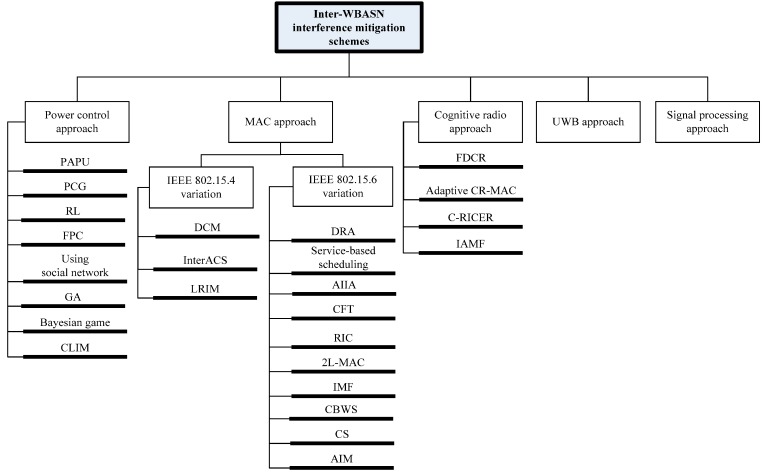
Categories of inter-WBASN interference mitigation schemes.

### 2.2. Inter-Domain Coexistence and Interference

Inter-domain coexistence and interference occur between many technologies that work in the same frequency at the same time and place. For example, WBASN, WiFi, and Zigbee devices may work within the 2.4 GHz band in one room. The performance degradation of WBASNs is investigated by experiment in [16]. The effect of other technology on WBASNs is explored with respect to the mathematical function of BER and symbol error rate. The simulation results show that WiFi negatively impacts WBASNs at the 6th, 7th, 8th, and 9th channels. The packet loss rate of WBASNs is high when there is a WiFi or Zigbee device within their transmission range.

## 3. Interference Mitigation Schemes

Inter-WBASN interference has been investigated in terms of its effect on the degradation of system performance. In this section, we review existing algorithms to mitigate inter-WBASN interference. The interference mitigation schemes can be categorized as shown in Figure 2.

### 3.1. Power Control Approach

Power consumption at sensor nodes in a WBASN is an important factor that affects the lifespan of the network. Because sensors are placed in or on the human body, it is necessary to reduce power consumption and save energy. As a result, interference mitigation schemes focus on power consumption in addition to the quality and throughput in an interference environment.

#### 3.1.1. Proactive Power Update

In [17], the proactive power update (PAPU) algorithm is presented as a new interference mitigation scheme to minimize power consumption and maximize throughput at each WBASN. At each WBASN’s coordinator, PAPU is applied by game theory to optimize the tradeoff between network utility and power efficiency. Game theory is applied as follows: A player is defined as a transmission link in one time slot, and an action of each player represents the transmission power and has a payoff function. The payoff function describes the tradeoff between the network utility and the cost function, with the cost function consisting of transmission power and power price. In this game theory, each WBASN maximizes its own payoff function to obtain the best response for each player. The best response is the transmission power of a player that maximizes its own payoff function.

Game theory applies to PAPU as in the following steps: At first, each player in a WBASN initializes its transmission power and broadcasts this information to its neighbor. Next, the player updates its neighbor’s transmission power and SINR. If the player detects the updated power from its neighbors, it calculates its best response and exchanges this information to the others. This algorithm is evaluated based on the unique Nash equilibrium.

#### 3.1.2. A Power Control Game: Nonlinear and Adaptive Power Pricing Function

In [18], the power control game (PCG) is an algorithm that, like PAPU, applies game theory to WBASNs. The main objective is to maximize the total system throughput, minimize power consumption, and penalize high power consumption.

In PCG, game theory can be applied as follows: A player is the transmission link within a WBASN at a certain time slot. An action of each player is as a set of transmission power levels that must satisfy the given power constraint set. The payoff function for each player is defined as the tradeoff between network utility and price function. The price function in [18] is more effective than the cost function in [17] because the price function in PCG can adapt to dynamic changes in channel gain or power budget. More specifically, the adaptive price function is clearly defined as a function of the channel gain, SINR, and power budget of each player. Therefore, the player has a higher chance to transmit if its power budget is high or its channel condition is good. Otherwise, its transmission is penalized to preserve energy and avoid interference from others.

Status updates within WBASNs in this algorithm are the same as in PAPU [17]. In each time slot, the player updates the transmit power to reach its best response in the next time slot. Consequently, this algorithm allows WBASNs to control their power level and maintain acceptable capacity for each user.

#### 3.1.3. Reinforcement Learning in Power Control Games

A power control algorithm called reinforcement learning (RL) in PCGs is reported to improve the performance of WBASN by a learning algorithm [19]. Although the control power in this algorithm is similar to PAPU [17] and PCG [18], it is distinctive due to its reinforcement learning algorithm. RL algorithm based on game theory considers *M* users in WBASNs as *M* RL-agents. Each RL-agent transmits at the maximum data rate, and RL’s payoff function is considered as a reward function to choose the best-responding player. The reward function is updated at each state of network changes.

#### 3.1.4. Fast Converging Fuzzy Power Controller

In [20], the fast converging fuzzy power controller (FPC) system model is described as a fuzzy system with a feedback channel to minimize transmission power and maximize link throughput. Genetic algorithm (GA) is applied to determine the optimal fuzzy controller parameters.

The system model contains the inputs, output, and fuzzy power controller. The input signals are SINR, current interference power, and a feedback signal. The output is the current transmission power, which works as the feedback channel to the input of the system. The fuzzy power controller decides on the level of transmission power at the output based on its inputs. The fuzzy power controller consists of a fuzzy knowledge base, crisp input, crisp output, fuzzifier, fuzzy interference engine, and defuzzifier.

GA is employed at the fuzzy knowledge base to control the fuzzy interference engine. GA learning mechanism is used to describe the fuzzy rules and the output variables to create suitable parameters for the design criteria. GA is divided into four stages: The first stage is called the chromosomes where parametric genes and rule genes represent the fuzzy set function of inputs and fuzzy logic rule, respectively. The second stage uses crossover and mutation operators to obtain new individuals. The third stage is called the learning strategy which chooses the best individuals. The last stage tries to maximize the fitness function that is represented to the link capacity, transmission power, and number of iterations needed to converge. More detailed information on the genetic-fuzzy system can be found in [20].

#### 3.1.5. PCG Using Social Networks

An algorithm to mitigate interference in a cyber-physical WBASN system was designed by detecting network topology and applying PCG [21]. In a scenario with people wearing WBASNs, changing their positions changed the network topology, thereby causing interference with other WBASNs. This mechanism includes two algorithms: the power control game and the social contact network consisting of WBASNs.

In the social contact network consisting of WBASNs, a tool for social networks was devised to build a social interaction network. An interference graph of the overlapped communication range was created in which the vertices were the nodes and the edges were the links between two adjacent nodes. The detection of dynamic interaction in social networks was developed using Bluetooth and acoustic wave technology. The distance between two WBASNs was detected by sending and receiving an acoustic wave, and the WBASN calculated the time delay to estimate the distance between them. A four-state dynamic social interaction prediction algorithm was used to detect the interaction in dynamic topology by applying Markov Chain. The four states of the predictor can be listed as strong interaction, weak interaction, strong noninteraction, and weak noninteraction. Moreover, the channel gain model was developed for three-tier network topology, which is same as in [9]. The channel gain is the function of the distance between the transmitter and the receiver in two different tiers.

The second algorithm is the PCG based on the output parameters in the first algorithm. Similar to the PCG in [17,18], the network utility and the transmit power are optimized. The player in this game is the communication link between the transmitter and the corresponding receiver. The SINR at each node is calculated by using the channel gain in the first algorithm. The price value of each player is defined as the function of the SINR, weight, and transmission power. The weight is set according to WBASNs’ wireless channel state, power state, constraints on power, and quality of service (QoS) requirements. In the PCG, each WBASN estimates the distance and predicts social interaction among people; then the WBASN updates its power and price values.

#### 3.1.6. Power Allocation Using GA

In [22], a power allocation algorithm is designed to mitigate interference while guaranteeing the QoS requirements. In this algorithm, an optimization model is used to minimize the transmission power of the sensor in a WBASN by satisfying the required transmission power under the constraint of sensor QoS requirements. The GA is applied to WBASNs to solve the optimization problem. The model for GA is a biological evolutionary process in which the individual of the population is the sensor power and the fitness function is the optimal power allocation strategy with minimum total power under QoS constraints. The following steps are used to solve that problem: (1) Initialize the individual in the generation; (2) evaluate the fitness function; (3) gene selection; (4) crossover process; (5) mutation process; and (6) evaluate the fitness value. Further information can be found in [22].

#### 3.1.7. Bayesian Game Power Control Scheme

Another PCG based on the Bayesian game is used to mitigate inter-WBASN interference [23]. This system is modeled with multiple WBASNs in which each WBASN consists of one coordinator and multiple sensors. The Bayesian game is defined as follows: A player is a WBASN; the type set of players is identical to the node set that is scheduled to transmit; the set of players’ actions chooses its power; the payoff function for players includes the type and the corresponding power. In this scheme, the WBASN tries to maximize its payoff that is evaluated by the Bayesian equilibrium. Each WBASN maps to the set of actions based on its type.

#### 3.1.8. Cross-Layer Interference Management

Another power control scheme is the cross-layer interference management (CLIM) which defines the interference-limited communication range around the receiver [24]. The transmission and interference range are modeled as a disk graph. The interference-limited communication range is calculated in two cases of the path loss exponent while ensuring successful communication links. If a WBASN receives interference from others, it adapts its transmission rate and power to reduce interference. The network performance is maximized in both physical and medium-access layers because concurrent transmissions are allowed.

### 3.2. MAC Approach

Interference between WBASNs can be mitigated by switching their working channel or manually scheduling their superframe. In this approach, a MAC protocol is explored to maximize throughput and minimize transmission power while mitigating interference.

#### 3.2.1. Dynamic Coexistence Management

Dynamic coexistence management (DCM) [25] is a new MAC scheduling algorithm designed to manage the coexistence of WBASNs based on the IEEE 802.15.4 standard [5]. WBASNs with DCM are able to detect and mitigate the effects of coexistence independently. A scenario in which many WBASNs transmit simultaneously may cause beacon collision and data collision [11]; DCM uses beacon replacement and channel switching, respectively, to resolve these collisions.

Because clear channel assessment (CCA) is not used in beacon transmission, beacon collision prevents the sensors from transmitting to the superframe. At the end of a contention-free period (CFP) in the superframe, the coordinator can detect a lost beacon. If a single beacon is lost, the coordinator searches the current channel for the inactive part of superframe. The beacon will resume normal activity if the coordinator concludes that the beacon was lost only once. In contrast, the beacon must be replaced on the timeline if the loss occurred at the second superframe or frames from coexisting WBASNs are detected. When a beacon loss is detected, the coordinator scans the current working channel during the beacon interval (BI) of the superframe to decide when to transmit a beacon. There are two cases: The coordinator will transmit a beacon in a gap of its own period, or it will replace the beacon at the largest available gap.

In data transmission, the coordinator detects lost data at the end of the CFP. Whether these collided frames are retransmitted or not is based on acknowledged or unacknowledged mode, respectively. In the DCM scheme, the coordinator scans a candidate channel for a possible channel switch. The two possible options are the full BI scan and the inactive period scan, which are described as follows. In the case of the full BI scan, the coordinator will have information about the preexisting WBASNs in the candidate channel and the available gap for its own period. In case of the inactive period scan, the coordinator scans the channel to find a gap without missing a beacon, even though the information about the preexisting candidate channel is not available. In the case of an unsuccessful scan for a candidate channel, other channels will be monitored during the inactive parts of the next superframes. If there is no inactive period in superframe (SD = BI), the coordinator will search for the whole BI.

#### 3.2.2. Interference-Aware Channel Switching

Interference between WBASNs can be monitored by using an interference framework that decides to switch the operating channel of interfering WBASNs to another channel [26]. An interference framework includes a set of network nodes, a set of device state models, and the interface interconnecting them. The interference framework is able to record the duration of nodes and notify the current state of the node. In the Interference-aware Channel Switching (InterACS) algorithm, the signal-to-interference ratio (SIR) of the sender is monitored by the coordinator. If the SIR is below a threshold value, the coordinator switches the sender to a new channel. A threshold value is in the range of three values: low, moderate, and high; in these cases, the coordinator switches the sender to the next 2-hop channel, 3-hop channel, and 4-hop channel, respectively.

#### 3.2.3. Lightweight and Robust Interference Mitigation Scheme

A new interference detection method based on the estimation of beacon delivery ratio (BDR) is lightweight and robust interference mitigation scheme (LRIM) which is introduced in [27]. The transmission efficiency (TE) is a function of successfully received packets, the number of backoffs, and the number of retransmissions. The threshold values of TE and BDR are used to indicate the severity of interference in WBASNs. The coordinator makes decisions on adaptive channel hopping based on TE and the channel hopping requests from its sensors. If more than half the sensors send the request, the coordinator decides to hop channels by selecting a watcher. The watcher, a node with high RSSI and low traffic load in a WBASN, senses the channel status and sends the result to the coordinator. When the coordinator discovers an idle channel, it will hop the operating WBASN to that channel.

#### 3.2.4. Dynamic Resource Allocation

In [28], dynamic resource allocation (DRA) is presented as a new MAC algorithm that aims to achieve spatial reuse while avoiding interference among multiple WBASNs. Each WBASN creates a list of interfering sensors from other WBASNs within its transmission range and broadcasts this list to its neighbors. Each coordinator will assign channels to the interfering sensors orthogonally.

The interference scenario was investigated in a system of many WBASNs with numerous sensors in each WBASN. Each WBASN’s coordinator knows the received power of its sensor nodes. This algorithm starts with the orthogonal transmission of sensor nodes of each WBASN in the first round. The received power of each sensor is compared to the SINR threshold; an interference list is then created that consists of the sensors in the interference range between WBASNs. Each coordinator then creates its interference set that consists of its interfered sensors and the interfering sensors belonging to its neighbors. All coordinators broadcast their interference sets and assign new channels to the nodes with a high interference level.

**Figure 3:**
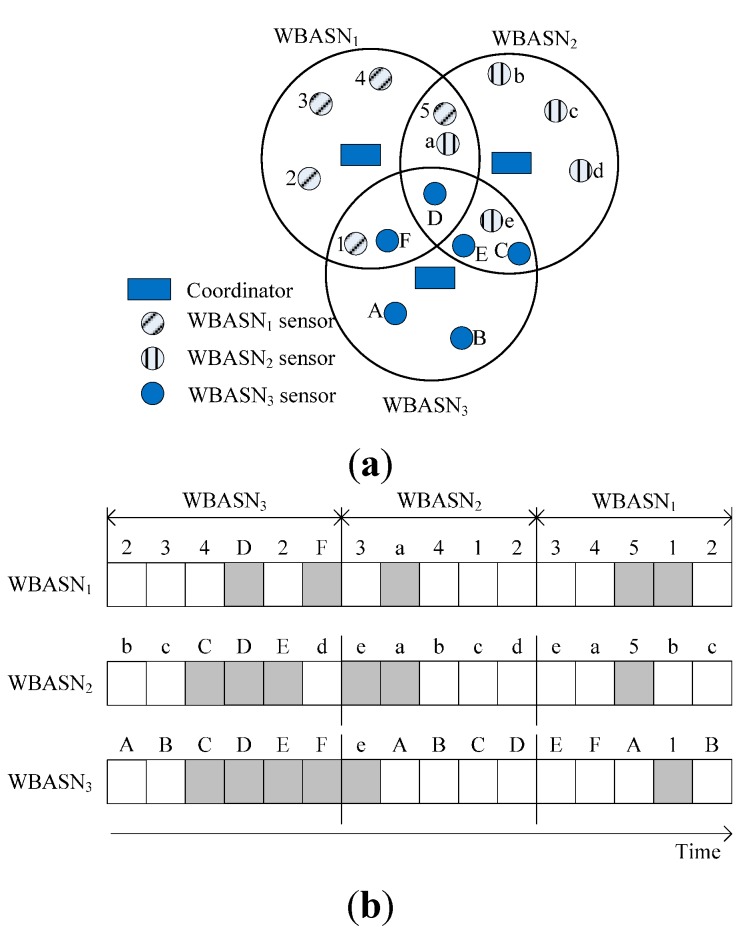
(**a**) An example of interference among WBASNs; and (**b**) channel assignment in DRA.

An example of channel assignment is shown in Figure 3a with three coexisting WBASNs labeled WBASN_1_, WBASN_2_, and WBASN_3_. Each WBASN will create its interference list as follow, I1 = {(2, a), (3, F), (3, D)}, I2 = {(1, 5), (3, C), (3, D), (3, E)}, and I3 = {(1, 1), (2, e)} for WBASN_1_, WBASN_2_, and WBASN_3_, respectively. The interference set is also created at each coordinator as follows: S1 = {(1, 1), (1, 5), (2, a), (3, F), (3, D)}, S2 = {(1, 5), (2, a), (2, e), (3, C), (3, D), (3, E)}, and S3 = {(1, 1), (2, e), (3, C), (3, D), (3, E), (3, F)}. The channel assignment is shown in Figure 3b; nodes in the interference set are transmitted orthogonally.

#### 3.2.5. Service-Based Scheduling

In [29], a QoS-based MAC scheduling scheme is derived for multiple coexisting WBASNs in a scenario for healthcare monitoring. The interfering coordinators exchange information before transmitting data; thus, the coordinator will decide which sensor can access the channel. The channel is divided into beacon periods or MAC superframes. At each WBASN, the coordinator sorts the network traffic based on the priorities established in IEEE 802.15.6 [1] then assigns slots descending to the CFP. The user with the highest priority is assigned to the first available slot. In the first beacon, the coordinator broadcasts WBASN identification and timer information. If other coordinators are within the transmission range and listen to this schedule table, they will save this information in their respective tables. In the case of overlapped frequency range and assignment of transmission slot, the priority level of slots in the interference range will be compared, and the lower-priority slot will be delayed to transmit later. In the next beacon, the coordinator broadcasts the traffic information to all devices. Devices know when to transmit in the next superframe, thereby avoiding inter-WBASN interference.

#### 3.2.6. Asynchronous Inter-Network Interference Avoidance

In [30], a hybrid multiple-access algorithm of CSMA/CA and TDMA is used in a new MAC algorithm, asynchronous inter-network interference avoidance (AIIA). AIIA is time-independent synchronous algorithm to avoid interference by listening to the controlling message from other WBASNs then updating its own schedule.

**Figure 4:**
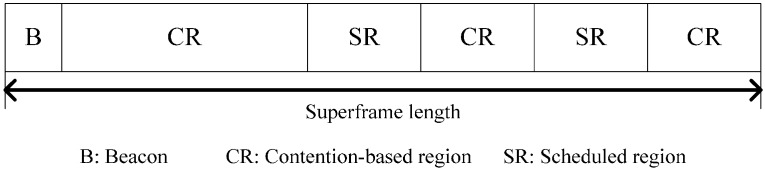
Superframe structure in AIIA.

The superframe structure of AIIA is shown in Figure 4 and includes the contention-based regions (CR) and the scheduled regions (SR). The differences between AIIA superframe and IEEE 802.15.6 superframe [1] are: (1) The CR can correspond to the EAP, RAP, and CAP periods in IEEE 802.15.6, where CSMA/CA is used for resource allocation; (2) the SR can be splitted into multiple periods in AIIA that are similar to the MAP periods in IEEE 802.15.6, and TDMA is used in SR.

For each WBASN, the gateway device or coordinator runs AIIA to maintain two components: overlapping WBASN detection (OWD) and scheduled region relocation (SRR). The sequential operations of these components are described as follows.

In the first operation, OWD is repeated periodically at the gateway to maintain the AIIA table. The scheduled region advertisement (SR_AD) message, which includes the AIIA table entries and the clock timer information, is created at every gateway to exchange information with the others. When many WBASNs are working at the same operating channel, the gateway of each WBASN broadcasts its SR_AD message before the start time of its SR period and listens to the SR_AD messages from the others. The gateway then constructs the AIIA table, which includes six fields. The first field is the gateway identifier (*GWID*) field to store the received device identifier. The second field is the *hop-count* field to indicate the number of hops from the gateway to a neighboring gateway. The next field is the *superframe_offset* to store the time difference between the GW’s timer and a neighboring GW’s timer. The *TdmaStarttime* and the *TdmaDuration* fields indicate the start time and the duration of SR period, respectively. The last field, *SeqNo*, indicates the latest version of the corresponding entries.

**Figure 5:**
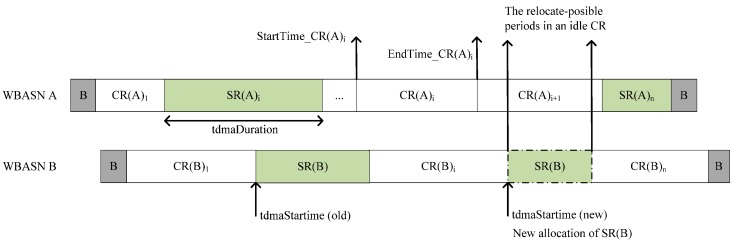
An example of the SRR operation.

In the second operation, SRR, the gateway reschedules its conflicting SR to another location within the CR period of the other WBASNs. SRR consists of three phases. Phase I is the “searching for an idle period in CR” phase in which the gateway searches for the CR periods by referring to its own AIIA table, remembers the start and end times of the CR, then relocates the conflicting SR period and transmits the SR_AD message. In phase II, the gateway finds the relocate-possible (RP) periods amongst the CRs of other WBASNs and assigns the indices to RP and remembers the location based on the index of the CR. In phase III, the gateway determines the new location of the SR period and the transmission time of the SR_AD message and, then the changed SR schedule is broadcasted to the other gateways of its neighbor WBASNs. An example of the SRR operation is shown in Figure 5.

#### 3.2.7. Continuous Frame Transmission

Continuous frame transmission (CFT) is a method to reduce the energy consumption in an interference environment involving remote and home healthcare system applications [31]. The CFT MAC protocol is based on the TDMA implemented in ultra-low-power devices. CFT utilizes carrier sensing to control each transmission time to avoid signal interference from nearby devices. The medium inter-frame space (MIFS) indicates recognition of interference. If a device detects interference during a node’s transmission, it suspends its transmission and retransmits at the end of the final interfering frame. I_SIFS is the inter-frame space between consecutive frames sent by one node. I_LIFS is sent from devices in the network to avoid frame collision. If IDLE time occurs longer than MIFS after a frame transmission, MIFS indicates that the interfering frame is over, whereas a shorter IDLE time indicates that the interfering frame is the next frame.

The CFT method is implemented as follows: If devices sense that the channel status is IDLE, they start to transmit. Otherwise, they wait until the channel status becomes IDLE and, then, they wait for a MIFS interval and transmit the next frame. MIFS is determined by the value of I_SIFS and I_LIFS.

#### 3.2.8. Random Incomplete Coloring

In [32], *inter*-WBASN interference is mitigated by considering inter-WBASN scheduling (IWS) as a problem of graph coloring. The graph coloring problem aims to achieve a tradeoff between spatial reuse and convergence.

WBASNs consist of one central processing node (CPN) and wireless sensor nodes (WSN). A unit-disk graph in RIC is generated with the vertices representing the set of CPNs and the edges representing the conflict links between CPNs. This model is called CPN-based IWS.

**Figure 6:**
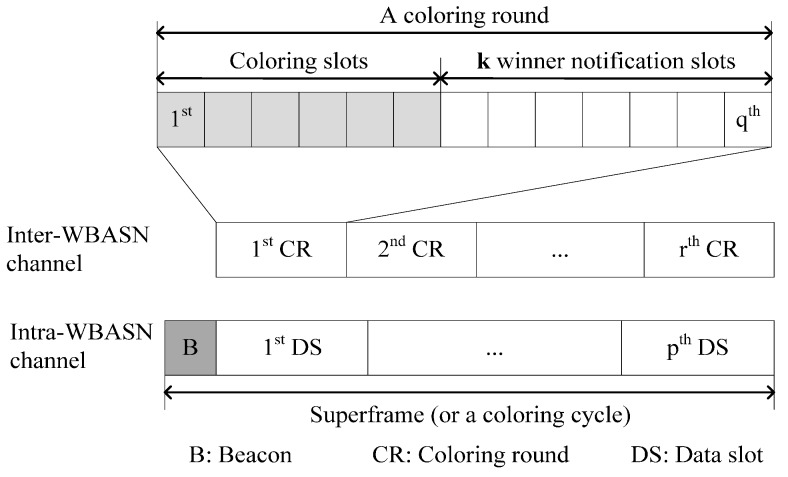
The superframe in RIC.

In random incomplete coloring (RIC), TDMA is used in both intra- and inter-WBASN channels. A superframe is equivalent to a coloring cycle in RIC. For intra-WBASN channels, the superframe consists of one beacon slot and *p* data slots. For inter-WBASN channels, there are *r* coloring rounds in one coloring cycle in which each coloring round is divided into coloring slots and winner notification slots. The superframe in RIC is shown in Figure 6.

The RIC-coloring algorithm is an oriented coloring in which two nodes in one edge are assigned different colors. Each vertex in graph coloring adopts a random value from an available color set, and then, CPN or the vertex broadcasts the coloring message to other vertices. This message contains the color and the random value information. If any vertex receives a message that has the same the color and a higher random value, the vertex is uncolored; otherwise, it wins that color. When a vertex wins the color, it will send this information to all vertices.

The two steps of IWS for CPNs are the negotiation between CPNs and the broadcast, which use the inter-WBASN channel and intra-WBASN channel, respectively. In the first step, CPNs randomly choose a color in every coloring round, and then, they exchange the coloring message through the coloring slots. In the second step, if the CPN wins the slot, it will broadcast the information on the winner notification slot and send a beacon to its WSNs to indicate the time slot for the WBASN. The number of data slots that can be scheduled in each coloring cycle is indicated as the winner notification slots or the colored nodes. The remaining nodes that are not colored suspend transmission to avoid interference; they then may be activated in the next round.

#### 3.2.9. Two-Layer MAC

Two-layer MAC (2L-MAC) is the two-layer interference mitigation for WBASNs to ensure that the QoS requirement is met [33]. The algorithm is described in two phases: polling with backoff mechanism and channel switching.

In this algorithm, the hub of a WBASN always checks for the idle channel before polling its sensor nodes. It then assigns the channel to the sensor nodes for intra-WBASN traffic by TDMA-based polling. Inter-WBASN interference can be avoided by a carrier-sensing mechanism between hubs. The QoS requirement is defined in the IEEE 802.15.6 standard as well as the contention window (CW) and backoff timer (BT) [1].

**Figure 7:**
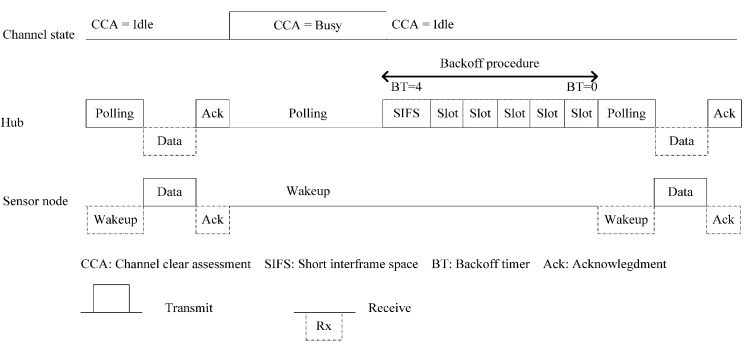
Polling with a backoff mechanism in 2L-MAC.

In the polling with backoff mechanism, the hub performs carrier-sensing CCA to check the channel state before sending an intra-WBASN poll frame. In case 1, the channel is idle, and the hub sends a polling frame to the sensor nodes. When nodes receive the polling frame, they send data to the hub within the SIFS period. In case 2, the channel is busy, and the hub performs the backoff procedure and waits until the channel is idle. The hub knows the priority of each node in the WBASN and therefore sets the CW size based on the priority value. The hub chooses a value of BT and waits until the channel becomes idle. When the hub senses the idle channel status, the BT is decreased by one in each time slot. When the BT is zero, the hub sends a polling frame to the sensor node and waits for data from its sensor nodes. Figure 7 shows an example of the polling with a backoff mechanism.

In the channel switching mechanism, each sensor must wait for a delay time of *T_delay_* that is the time period from when its wake up event is triggered to when it receives a polling frame from the hub. If *T_delay_* is longer than the predefined threshold *H_switch_*, both the hub and the sensor switch from the current operating channel to a backup channel; otherwise, a backup channel is chosen by the hub at the previous polling frame. A WBASN performs channel switching based on a channel sequence that is calculated using a 16-bit Galois linear feedback shift register function defined in [1].

#### 3.2.10. Interference Mitigation Factor

In [34], the interference mitigation factor (IMF) is a quantitative measure of adaptive mechanism for mitigating interference among multiple WBASNs. The SINR value in a WBASN is used to maintain link quality and adapt different schemes such as modulation, data rate, and duty cycle. In this mechanism, the IMF determines the reduced transmit power level in the adaptive transmission status. Then, each WBASN selects its modulation scheme, data rate, and duty cycle value based on the IMF.

For adaptive modulation, the set of modulation schemes is BPSK, QPSK, and 8PSK. If the link quality is good or the SINR is higher than the threshold SINR, WBASN chooses 8PSK modulation for transmission. Otherwise, BPSK modulation is chosen, indicating that the link quality is not good. The data rate and the duty cycle can also be adaptively set, and they have the maximum values at normal operating mode. In addition, the IMF is calculated on a case-by-case basis: (1) adaptive modulation, in which the IMF is the function of the SINR and channel condition; (2) adaptive data rate, in which the IMF is the proportional function of data rate and transmitted power; and (3) adaptive duty cycle, in which the IMF is the function of the duty cycle in normal state and current state.

#### 3.2.11. Clique-Based WBASN Scheduling

In [35], node-level interference is explored with respect to the type of sensor nodes in one or more WBASNs. Because sensors can be divided into different types that send data periodically or nonperiodically, all sensors in a WBASN may not be active at the same time. Because of the mobility of WBASNs, sensors in different WBASNs can be active simultaneously, causing interference. Clique-based WBASN scheduling (CBWS) places the sensors in different groups to avoid interference then schedules the sensors by applying the coloring method.

In the system model, a clique can be formed by one or more WBASNs using a distributed method. The sensor nodes in the clique are grouped into virtual groups based on their group IDs. The sensors in a group will be allocated into time slots by the random coloring algorithm. The WBASN network topology is modeled as a graph in which the vertices represent the set of groups and the edges represent the links between groups. The color set represents the resources such as time slots or frequency bands. The color mapping algorithm assigns the vertices to the color set so that the sensors can work in different time slots. After that, each group can work during the assigned time slot.

#### 3.2.12. Cooperative Scheduling with Graph Coloring

Cooperative scheduling (CS) with graph coloring [36] is the hybrid interference mitigation of the scheduling scheme and graph coloring. The scheduling scheme is similar to DRA [28], in which the sensors within a region affected by interference will transmit orthogonally. In CS, two nearby interfering WBASNs form one cluster. In each cluster, the sensor nodes will transmit orthogonally. The coloring method is applied to assign colors to every cluster so that every cluster has a different color transmitting simultaneously. Interference avoidance is modeled as a graph in which the vertices represent the wireless nodes and the edges represent the resource collision. The color set comprises the time slots that will be assigned to the vertices.

#### 3.2.13. Adaptive Internetwork Interference Mitigation

Similar to DRA [28] and CS [36], the adaptive internetwork interference mitigation (AIM) algorithm [37] aims to increase spatial reuse. AIM reschedules the sensors of the interfering WBASNs into synchronous and parallel transmission. The priority of sensor nodes is used to assign time slots. The node with higher priority will be assigned more time slots. In the meantime, the other nodes in the transmission range suspend their transmissions.

AIM can be described in terms of three phases: The first phase is the orthogonal transmission in which the coordinator of a WBASN calculates the interference level based on the sensor node’s information such as received power, packet length, and priority level. The sensor nodes are then assigned orthogonal channels by exchanging messages from the coordinators between WBASNs. In the second phase, the interference list of each WBASN is formed, similarly to DRA [28]. Next is the information exchange phase in which every coordinator broadcasts its interference list. Finally, WBASNs schedule their transmissions based on the traffic priority. The simulation results show that AIM without considering priority achieves lower efficiency than AIM considering priority.

### 3.3. Cognitive Radio Approach

#### 3.3.1. Fast Dynamic Cognitive Radio

In [38], the fast dynamic cognitive radio (FDCR) algorithm is implemented in adaptive cognitive enhanced platform (ACEP) to schedule access for a WBASN’s node to the idle spectrum. ACEP is designed according to CR-WBASN models and an interference subsystem, an observer subsystem, and a WBASN subsystem. The function of each subsystem is as follows: An interference subsystem can be considered as the existence of WiFi interference in a medical environment. An observer subsystem consists of two observation nodes to monitor the whole system’s working status. The coordinator in the WBASN subsystem, which is implemented with FDCR, senses the spectrum and estimates interference behavior patterns.

In an ACEP system, a MAC algorithm based on the IEEE 802.15.6 standard gives the coordinator the ability to sense the spectrum and estimate interference behavior patterns. FDCR then adjusts the channel access and sensing times based on the spectrum occupancy condition, thereby increasing the stability of the WBASN’s access to channels.

The FDCR algorithm can be divided into two phases: channel sensing with sensing time *T_s_* and channel access with access time *T_a_*. If the sensing time is short and the access time is long, the collision rate is low and the channel utilization is therefore good. In the channel sensing phase, the coordinator of a WBASN senses the channel based on the received signal strength indicator (RSSI). The coordinator indicates the spectrum status: idle or busy. In the channel access phase, the coordinator evaluates the channel condition by using the collision rate *η.* The packet collision rate is determined by the average packet collision rate during the last channel access phase. This value is used for comparison to the acceptable limit *η_threshold_* to decide the next channel access phase of the WBASN. The WBASN initializes the *T_s_* and *T_a_*. If the channel is idle, the coordinator broadcasts the necessary parameters to its nodes, enabling them to access the channel based on access time. To prepare for the next access phase, the overall system collision rate *η.* is calculated and *T_s_* and *T_a._* are updated.

#### 3.3.2. Adaptive Cognitive Radio MAC

In [39], an adaptive cognitive radio MAC algorithm is adopted on a hybrid cognitive validation platform (HCVP) to minimize WBASN interference. HCVP is a platform for cognitive radio evaluation and includes two parts. The lower part comprises the PHY and MAC layers based on real hardware, and the upper part comprises computer software that includes the cognitive radio algorithm, the network layer, and other layers. In HCVP, WBASN nodes are connected to a computer and the coordinator is associated with the cognitive radio software of the computer.

In HCVP, this algorithm is implemented at the coordinator of WBASN and includes spectrum sensing, channel estimation, and channel accessing. The RSSI is chosen to indicate the working status of the spectrum. Channel estimation is performed in cognitive radio software, which results in the prediction of channel usage and the optimization of access times. The second phase can be divided into two stages: In the first stage, RSSI is used to estimate channel usage distribution; in the second stage, the packet collision rate is used to indicate the channel status that is compared to the result of the first stage. This comparison creates an optimized tradeoff between throughput and sensing time. In the channel access phase, the coordinator senses the spectrum then obtains the result of an optimized access time and compares it to the collision rate threshold. If the channel is idle, the coordinator broadcasts a beacon with boundary information about a superframe setting according to an optimized access time. Nodes in WBASNs continuously wait for a beacon; if they receive a beacon, each node senses the spectrum for a small time before accessing the channel to avoid packet collision between WBASNs.

#### 3.3.3. Cognitive-Receiver Initiated CyclEd Receiver

A hospital may be the environment with the highest level of interference for WBASNs. Many people wearing WBASNs may be staying or walking about a hospital at any time. In [40], a cognitive framework is applied to WBASNs for avoiding interference in hospitals. The Cognitive-Receiver Initiated CyclEd Receiver (C-RICER) MAC protocol includes three main tasks: The first task is channel sensing, which senses the channel and builds a full interference map including the frequency channels. The second task is power adaptation, which reduces transmission power to reduce interference. The final task is channel adaptation, which enables the coordinator to switch to another channel.

#### 3.3.4. Interference-Aware Management Framework

As in [40], interference within a hospital is considered in [41]. Cognitive radio is employed in devices attached to the human body, and medical devices in the hospital are designated as the primary users. The interference-aware management framework (IAMF) is introduced as a cognitive framework that consists of the database, measurement, evaluation and update function, strategy function, and internal and external dissemination. The first task detects regions of overlap between WBASNs and calculates the position of mobile cognitive radio nodes. Power control and frequency hopping methods are then implemented in the cognitive radio platform to avoid interference.

#### 3.3.5. Cognitive Radio WBASN

The cognitive radio WBASN (CR-WBASN) model is based on UWB technology for medical applications in hospital environments [42]. The architecture model of the CR-WBASN consists of an IR-UWB transceiver with on-off keying modulation and an MB-OFDM transceiver. The frequency band is allocated to the UWB band (3.1 to 10.6 GHz). In [43], a three-tier WBASN architecture for hospital applications is introduced in which cognitive radio can be implemented in the WBASN controller node or the access point to send data to the server. A central controller-based architecture includes three components equipped with cognitive radio capabilities. Applying CR-WBASN can reduce electromagnetic interference and ensure QoS enhancement.

### 3.4. UWB Approach

In a UWB-based WBASN, multiple access interference (MAI) occurs in the transmission between devices on a single person. In [44], a successive interference cancellation (SIC) algorithm is introduced to cancel the interference. Also, the UWB/MIMO (multiple input/multiple output) system model is defined. A zero correlation duration code (ZCD) is applied to the UWB/MIMO WBASN system to spread the transmitted UWB signals. SIC orders the relative signals between the users based on the strongest signal. SIC determines the strongest signal to cancel and repeat until it achieves the desired signal with low probability of error. In this scheme, a transmitted signal is re-estimated at the receiver by using the interference cancellation scheme. At the receiver, zero forcing algorithm and minimum mean-square error algorithm are used to counteract the interference.

Another study focused on mitigating interference in a UWB body area network is conducted in [45]. A single hybrid hermit pulse is used to cancel the narrowband interference. The hermit pulse is shifted and modulated to create a hybrid hermit pulse. Because the hybrid pulses are designed to have notches at the desired bands, the narrowband interference is cancelled at the notches.

### 3.5. Signal Processing Approach

In the absence of interference in WBASNs, the signal processing of sensors only needs to rebuild the original signal and remove both noise and electromagnetic interference (EMI) from power lines. The first approach was to reconstruct the waveform of respiratory signals by processing single-channel ECG [46]. The empirical mode decomposition (EMD) in [46] aims to sum the components that approximates the original ECG signal. The EMD algorithm determines instrinsic oscillations by using the charateristics of data. Another approach is to combine ECG and EEG signals in the scenario of heathcare service [47].

In the presence of interference in WBASNs, each receiver has the ability to remove interference from other systems by either using detectors or changing the structure of the receiver. In [48], the interference in CDMA-based WBASN systems is rejected by using multiuser detection. The multiuser detectors have two different structures which are the decorrelator and the minimum mean square error (MMSE) receivers. The decorrelator has a decision metric to remove correlation, and the MMSE receiver minimizes the mean square error between the transmitted bits and the outputs of the transformation. The system is modeled by both the belt-head on-body transmission and an interference source from other WBASNs. The result of simulation shows that the detectors outperform the conventional receiver using match filters.

On the other hand, an ultra-low power wake-up receiver (WUR) [49] is implemented in WBASNs to comsume low power and avoid interference from wireless devices. In WUR, the transmission modes depend on the type of transmission. That is, the data transmission uses the transceiver’s Gaussian frequency shift keying (GFSK) and the wake up transmission uses Gaussian on-off keying (OOK) modulated by pulse width. The WUR filters interference by using the preamble detector.

## 4. Comparison and Discussion

The purpose of the mitigation schemes is to guarantee that WBASNs operate stably even in highly populated and interference-prone situations. In this section, the benefits and detriments of each approach will be compared and discussed.

### 4.1. Power Control Approach

PAPU [17] is considered to be the primary game-centric power control approach to mitigate interference among WBASNs. Because a distributed power control algorithm is used for WBASNs, each WBASN acts selfishly to maximize its own performance. However, each WBASN within the range of interference needs to exchange information about its current transmit power, channel gain, and interference power. Each transmission of WBASNs is decided by the payoff function, which consists of a power price and the current power transmission. If the power price is high, the network utilization and power efficiency in the WBASN are low. Hence, the tradeoff between network utilization and the power constraint for each user is needed. The update process must be fast for each WBASN to reach its stable state. The simulation result in [17] shows that the convergence time in PAPU is acceptable. The advantages of PAPU are the fast convergence time and the low power consumption in the WBASN achieved by using a low power price. However, the power price must be considered further because it affects the convergence and tradeoff. The disadvantage of PAPU is that it is not suitable for a real-world mobile scenario because of the fixed channel gain and interference gain. Moreover, the power price does not clearly show the QoS requirements of the transmitted signal from sensors.

Although the update status in the PCG algorithm [18] is similar to the PAPU algorithm, PCG is more efficient than PAPU because PCG considers the dynamic environment problem by using an adaptive power price. In addition, the power budget of a WBASN is added to the tradeoff in the PCG. Therefore, the player in PCG is penalized if its power budget is low or its current channel status is bad. This scheme can mitigate interference in WBASNs and increase the performance of WBASNs in the varying channel scenario. Although PCG was developed to cope with a dynamic environment, it has some disadvantages. WBASNs must negotiate the best response for each network. This may lead to a long convergence time. Moreover, the QoS requirement of sensors is not considered in PCG.

Unlike PAPU and nonlinear PCG, a PCG with a self-learning strategy [19] allows a WBASN to act as a single-action learner agent that learns from its experience to reduce interference from other networks. As each WBASN employs an RL algorithm to improve its performance, the WBASN updates its next state based on the previous state and the current SINR value. The improvement in RL is that WBASNs do not negotiate with others, although this algorithm has been developed for the PCG. Another benefit is that users operate well under a dynamic environment (e.g., channel, power levels) by setting a learning rate for WBASNs. Furthermore, RL results in a better tradeoff than PAPU or basic PCG despite its long convergence time. However, as in PAPU and nonlinear PCG, the QoS constraint is not considered.

A novel genetic-fuzzy power controller [20] is a method that combines GA and FPC into a power control method. The benefit of FPC is similar to that of RL-based algorithms in that WBASNs do not need to negotiate with others. A new development in FPC is to employ GA to speed up the convergence time by using the current transmit power as a feedback value. Moreover, FPC uses this feedback value to improve the fitness function. The simulation result shows that the performance of FPC outperforms that of PCG [18]. Hence, it is reasonable to adapt FPC in a mobility scenario in which interference is unpredictable because FPC provides an independent and fast convergence method. It is clear that FPC is more effective than PCG and PAPU. Nevertheless, the QoS constraint of sensors is not mentioned here.

In [21], the interaction information between WBASNs is explored in terms of the cumulative distribution function of the distance between WBASNs. Based on that study, WBASNs detect the distance between others within their transmission range before broadcasting to every WBASN within the interference range. The first advantage of this comes from the interaction steps; the WBASN that receives this information needs to optimize its power to avoid interference. Another advantage is that a WBASN can update the changing network topology to adapt its transmit power. Despite that, the time of social information detection and convergence must be considered. Because WBASNs must exchange information about its own channel, SINR, network utilization, and channel gain before the update, these data must be detected fast and accurately.

GA [22], a new power control-based algorithm, considers the QoS requirement to be the major factor for improving WBASN service and reducing power consumption. QoS requirements are considered in two services. The first service is the throughput-sensitive service, which must guarantee the minimum throughput requirement of the sensor. The second service is the BER-sensitive service, which must know the maximum BER threshold of each sensor. The simulation in [22] shows that GA outperforms PAPU in terms of throughput and BER. The disadvantage of GA is that real-world mobility environments are not considered, which may lead to a slow update in the WBASN and a long convergence time.

The Bayesian game [23] also considers the tradeoff between power consumption and throughput, which is introduced in PAPU [17] and PCG [18]. While the other power control games do not take the sensor nodes into account, the Bayesian game schedules each sensor node’s transmission in its channel based on the type of sensors. Therefore, WBASN can adapt its power at the node level based on the information about the dynamic environment or the type of nodes. The main advantage of the Bayesian game is that it works without exchanging data between WBASNs. However, the severity of the interference at each sensor node is not considered, nor is the convergence time.

**Table 1:** Comparison of the interference mitigation schemes in the power control approach.

Interference Mitigation Scheme	Throughput	Energy Consumption	Mobility Support	Negotiation	QoS Guarantees	Self-Learning Ability	Channel Parameter	Convergence Time	Tradeoff
PAPU ^1^ [17]	Low	High	No	Yes	No	No	Channel gain, power, interference gain	Slow	Low
PCG ^2^ [18]	Low	Medium	Yes	Yes	No	No	Power budget, SINR	Slow	Low
RL-based ^3^ [19]	High	Medium	Yes	No	No	Yes	SINR	Slow	High
FPC ^4^ [20]	High	Medium	Yes	No	No	Yes	Power, SINR, power feedback	Fast	High
Using social networks [21]	Medium	High	Yes	Yes	No	No	Interaction information, channel gain, power, interference gain	Medium	Medium
GA ^5^ [22]	High	Medium	No	Yes	Yes	No	Channel gain, power, interference gain	Slow	Medium
Bayesian game [23]	High	High	Yes	No	Yes	No	Channel gain, power, interference gain	Slow	Low
CLIM ^6^ [24]	N/A ^7^	Low	No	No	No	No	Channel gain, SINR	N/A	N/A

^1^ Proactive Power Update; ^2^ Power Control Game; ^3^ Reinforcement Learning-based; ^4^ Fast converging fuzzy Power Controller; ^5^Genetic Algorithm; ^6^ Cross-Layer Interference Management; ^7^ Not Applicable.

In [24], cross-layer interference management attempts to reduce the power while ensuring the performance of the transmission. Compared to the other power control mechanisms, it is simple but effective to apply to WBASNs in a hospital environment. Because of its reduced transmission range, it cannot interfere with other WBASNs. However, the QoS constraint of sensor nodes is not considered. The above power control algorithms use TDMA; therefore, new MAC protocols that are suitable for IEEE 802.15.6 requirements must be established. A detailed comparison of these power control schemes is presented in Table 1.

### 4.2. MAC Approach

In an interference scenario, the DCM algorithm [25] first detects collisions between beacons or data so that the WBASN can evaluate the degradation of performance before switching the channel. Each WBASN’s coordinator must perform the channel scanning and data retransmission. This is an advantage of DCM, because WBASNs manage their own working status independently. Another advantage is the low latency of the data frame. However, this does not guarantee that the QoS requirement is met. Although the likelihood of successful transmission is high, the high power consumption of WBASNs may lead to increased temperatures of the sensors in WBASNs.

Unlike DCM, InterACS applies only the switching channel into WBASNs to mitigate interference without considering packet loss. In InterACS [26], the framework for interference records the node’s activity and status, and the coordinator selects the channel based on the current SIR value to avoid collision. Although this algorithm is simple and standard, it ensures inter-WBASN interference. However, the simulation in [26] was run with low-density WBASNs. Therefore, it is necessary to investigate the performance of InterACS in a high-density WBASN scenario. Moreover, InterACS does not consider a dynamic interference scenario, which will be faced in any real-world setting. Some requirements to consider are the power consumption, QoS constraint, mobility support, and end-to-end delay.

It has been shown by experimentation that the transmission efficiency of the lightweight and robust interference mitigation scheme (LRIM) [27] is suitable for application. As with InterACS [26], LRIM applies channel hopping to avoid interference. However, LRIM is more effective than InterACS because of channel sensing. Because LRIM employs a node as a watcher, the efficiency of channel sensing is high without affecting the performance of the whole network. Nevertheless, the mobility of WBASNs and the QoS constraint are not considered. Moreover, the end-to-end delay may be high because of the channel sensing and switching tasks.

The DRA scheme [28] considers the interfering nodes for rescheduling transmissions among WBASNs. Each WBASN within the interference range generates its own table of interfering nodes in every WBASN. WBASNs must negotiate before reallocating their time slots based on the knowledge of inter-network interference. DRA increases spatial reuse compared to conventional orthogonal channel assignment. This scheme, which investigates the shadowing effect and threshold value of the SINR, aims to ensure high performance of DRA with regard to the fading effect caused by human mobility. However, WBASN requires a low SINR threshold and must update its information upon every change in the network based on SINR. The convergence time depends on the broadcast at each round. Therefore, a correct decision between SINR and spatial reuse must be reached.

In the service-based scheduling scheme [29], the QoS requirement of traffic is the primary factor of concern regarding the allocation of data to the superframe in an interference scenario. The superframe is defined in IEEE 802.15.6 [1] and is used with beacon mode. Therefore, WBASNs with higher priority can transmit data in a collision scenario while the others suspend their transmissions. Moreover, the power consumption is not affected because the packets do not collide. The disadvantage of this scheduling scheme is that it does not consider dynamic environments, changing topology, or high-density WBASNs. The end-to-end delay is high for WBASNs in listening mode whereas the end-to-end delay is low for high-priority WBASNs.

The AIIA scheme in [30] also updates information by using a table. The AIIA table contains information about the location of the contention-free period in the superframe of neighboring WBASNs. The AIIA learns about the timing offsets between its timer and a neighboring GW’s timer through the coordinator and, then a TDMA transmission schedule is designated for neighboring WBASNs. Therefore, in scenarios of context-aware mobility, AIIA works well because it can transmit data to its neighbors without synchronizing between WBASNs. AIIA reallocates time slots to mitigate interference without negotiating with other WBASNs. The higher-capacity WBASNs and lower energy consumption are shown in the AIIA simulation results. However, the QoS of sensors is not considered in this algorithm, nor is the convergence time of the constructing table. In addition, there is a tradeoff between the length of the TDMA duration and the performance of the AIIA.

CFT [31], which uses a TDMA-based MAC protocol framework, can be considered as the simplest scheme. CFT is considered as a non-negotiation method which uses CCA for carrier sensing and schedules the time slot if the channel is idle. Due to the carrier sensing, the performance of CFT is better than TDMA. This method reduces unnecessary transmissions and is thus energy efficient. However, end-to-end delay and QoS are not considered in CFT. In a case involving interference between vital medical signals, retransmission may result in a long end-to-end delay.

The coloring method in [32] supports high spatial reuse in WBASNs. Convergence time is fast while power consumption is low, because the coordinators transmit a beacon to indicate which sensor can transmit. When a transmission is suspended, wasted energy is low. Despite these advantages, RIC does not consider the mobility of WBASNs or the QoS requirement of the vital signal in the superframe. Because this algorithm colors nodes randomly, the priority of each sensor in WBASNs is not considered. The node that transmits a vital signal may not be colored and therefore may not transmit, which could cause a dangerous situation in healthcare service scenarios.

The 2L-MAC scheme [33] is similar to service-based scheduling [29]. The QoS requirement is the primary factor that 2L-MAC uses to decide when to transmit a signal. The benefit of the cross-layer design is to avoid collisions at the physical layer and accessibility of the idle channel. A coordinator must scan for the idle channel then poll the channel with a back-off mechanism, which aims to prevent the polling from many hubs. Sensors switch to available channels, so the mechanism satisfies both the QoS requirement and the throughput of the WBASN. Nevertheless, the power consumption in the WBASN is high because the wake-up event at the sensor node is long. Also, the end-to-end delay is large because of the back-off mechanism.

Although adaptive techniques based on the IMF [34] are quite simple, they are acceptable applications for mitigating inter-WBASN interference. The SINR value is used to set a threshold for the switching technique. The advantage of the IMF is simply that because it is based on the standard MAC superframe, it can be widely applied to existing WBASNs. Nevertheless, the IMF mechanism which does not perform the channel scanning may cause collisions at the physical layer. In addition, the priority of the signal in WBASNs is not considered.

In [35], the main advantage of CBWS is that it considers interference at the node level with regard to the function of sensor nodes. In addition, the position of coordinators and sensors in WBASNs is considered for grouping sensors into a clique before reallocating them into time slots. Therefore, groups of the same type of sensors residing in the interference range can be rescheduled, resulting in a continuous communication link and reduced power consumption. Compared to RIC [32], CBWS applies the random coloring algorithm, but on the basis of the graph setup. In RIC, the colors are assigned to WBASNs so that the function of sensor nodes is not considered. In CBWS, only active sensors will be assigned colors, which leads to low power consumption and a long network lifespan while mitigating interference.

**Table 2:** Comparison of the interference mitigation schemes in the MAC approach.

Interference Mitigation Scheme	Throughput	Spatial Reuse	Collaborative Method	QoS Guarantees	Channel Parameters	Channel Access	End-to-End Delay	Number of WBASNs
DCM ^1^ [25]	High	No	No	No	Beacon, data loss detect	TDMA ^13^	Low	High
InterACS ^2^ [26]	Medium	Low	No	No	SINR	TDMA	High	Very low
LRIM ^3^ [27]	High	No	No	No	BDR ^18^, TE ^19^	CSMA/CA ^14^	High	Medium
DRA ^4^ [28]	High	High	Yes	No	SINR	TDMA	Medium	High
Service-based scheduling [29]	Medium	Medium	No	Yes	Transmit only sensing idle channel	TDMA	High	Low
AIIA ^5^ [30]	High	Medium	Yes	No	Superframe time offset	TDMA, CSMA/CA	High	Low
CFT ^6^ [31]	Medium	No	No	No	CCA ^15^	TDMA	High	Medium
RIC ^7^ [32]	Medium	Medium	Yes	No	Coloring message	TDMA	High	High
2L-MAC ^8^ [33]	High	No	No	Yes	SIFS ^16^ period	TDMA	High	Medium
IMF ^9^ [34]	High	No	No	No	SINR	TDMA	High	Low
CBWS ^10^ [35]	High	High	Yes	Yes	Group ID	TDMA	Medium	High
CS ^11^ [36]	High	High	Yes	No	SINR ^17^	TDMA	High	High
AIM ^12^ [37]	High	Low	Yes	Yes	SINR	TDMA	High	High

^1^ Dynamic Coexistence Management; ^2^ Interference-Aware Channel Switching; ^3^ Lightweight and Robust Interference Mitigation scheme; ^4^ Dynamic Resource Allocation; ^5^ Asynchronous Inter-network Interference Avoidance; ^6^ Continuous Frame Transmission; ^7^ Random Incomplete Coloring; ^8^ Two Layer MAC; ^9^ Interference Mitigation Factor; ^10^ Clique-Based WBASN Scheduling; ^11^ Cooperative Scheduling; ^12^ Adaptive Internetwork interference Mitigation; ^13^ Time Division Multiple Access; ^14^ Carrier Sensing Multiple Access with Collision Avoidance; ^15^ Clear Channel Assessment; ^16^ Short Inter-Frame Space; ^17^ Signal to Interference plus Noise Ratio; ^18^ Beacon delivery ratio; ^19^ Transmission Efficiency.

The AIM algorithm [37] combines the advantages of DRA [28] and the priority-based scheduling algorithm. The priority of each sensor node is considered for orthogonal scheduling. Therefore, the signal QoS constraint is ensured at the receiver. However, the end-to-end delay of signals is high because the sensor nodes can transmit in only one slot and then must wait for their turn.

The CS algorithm [36] applies the scheduling scheme and the graph coloring algorithm to mitigate interference at WBASN sensor nodes. The graph coloring algorithm is more effective than DRA [28]. According to the performance study, spatial reuse in the CS algorithm is high because clusters with different colors do not collide. However, the priority of the sensor nodes and the mobility of WBASNs are not considered. Mobility may lead to change in the network topology. As a result, the coloring algorithm and cluster formation may take more time to converge.

In summary, the purpose of MAC approach schemes is to reschedule the operating channel of the working WBASN to another channel in time or frequency domain. Some MAC algorithms are acceptable to mitigate interference. The throughput is the most important factor and has been thoroughly investigated to prove the efficiency of interference mitigation. In addition, some requirements are not explored in those schemes, including power consumption, QoS constraint, and end-to-end delay. System performance must be analyzed in terms of those requirements. A comparison of these MAC approach schemes is presented in Table 2.

### 4.3. Cognitive Radio Approach

Cognitive radio is a technique that can sense and access a channel as a secondary user. When a WBASN works in an interference scenario, smart channel selection combined with a suitable MAC is needed. Cognitive radio-based WBASN can improve system performance and avoid interference. With the cognitive radio scanning process, spectrum status is estimated, giving WBASNs more flexibility to change their operation.

In the adaptive cognitive radio MAC (CR-MAC) for HCVP [39], the sensing process is performed before the coordinator starts its transmission; therefore, packets do not collide. The accessing time is decided at the coordinator after it calculates the collision rate based on the received RSSI. The Contention Window value is set by the priority of the traffic, reducing the collision rate and thereby improving the throughput of the system. In this case, the idle listening in the sensor nodes may cause a long end-to-end delay and high power consumption.

The algorithm in FDCR [38] performs the same channel sensing operation as CR-MAC [39]. The RSSI is an important value to compare with the given threshold value, and the result is indicated to the idle spectrum or busy spectrum. One disadvantage of this scheme is that if beacon collision occurs and the WBASN senses the busy channel, the halted transmission may lead to a large end-to-end delay.

Unlike adaptive CR-MAC [39] and FDCR [38], the cognitive framework of C-RICER [40] aims to switch the operating WBASN to another channel. This is more effective because the interference can be mitigated and the WBASN operation is continuous without halting [39]. Nevertheless, the QoS constraint is not considered and the construction of an interference map may cause a long delay. Similarly, the framework in [41] performs channel hopping based on the detection of an overlapping interference region.

CR-WBASN is the model used for medical applications that requires a cognitive radio protocol for spectrum sensing [42,43]. The cognitive radio approach is considered as a promising technique to avoid both inter-WBASN and inter-domain interference. However, more research must be conducted for protocols in this model on such challenges as developing a MAC protocol for CR-WBASN that works with the IEEE 802.15.6 standard physical layer. A comparison of these cognitive radio approach schemes is presented in Table 3.

**Table 3:** Comparison of the interference mitigation schemes in the cognitive radio approach.

Interference Mitigation Scheme	Throughput	QoS Guarantee	Collaboration	Channel Parameter	Collision Rate
FDCR ^1^ [38]	High	No	Yes	RSSI^5^	Lower
Adaptive CR-MAC ^2^ [39]	Medium	Yes	Yes	RSSI	Low
C-RICER ^3^ [40]	N/A^4^	No	Yes	RSSI	N/A

^1^ Fast Dynamic Cognitive Radio; ^2^ Cognitive Radio-MAC; ^3^ Cognitive-Receiver Initiated CyclEd Receiver; ^4^ Not Applicable; ^5^ Received Signal Strength Indicator.

### 4.4. UWB Approach

UWB technology has been developed as one of the PHY specifications in the IEEE 802.15.6 standard. Although UWB-based WBASNs inherently have very low power density, interference between UWB transceivers or UWB systems occurs at the receiver [44]. The simulation result shows that ZCD is suitable for MAI and interference cancellation because the correlation period of ZCD is zero. Performance of systems employing SIC is better than non-SIC systems in terms of low bit error rate.

However, devices working in the narrow band can cause interference with UWB-based WBASNs. This type of interference can be cancelled by using pulses in WBASNs [45]. The simulation results show that the hybrid hermite pulses (HHP) system improves the bit error rate in cases of low SINR (high noise). The simulation also compares BER and SINR between the Gaussian pulse and the HHP system to show that WBASNs with HHP outperform those with the Gaussian pulse system.

### 4.5. Signal Processing Approach

CDMA is not considered as a promising scheduling technique to avoid interference between WBASNs [10]. However, in CDMA-based WBASNs combined with the multiuser detection, the interference from other systems can be mitigated and, thus, the BER performance of the on-body transmission is acceptable [48]. Another technique which is robust to interference is the WUR [49]. According to the simulation results, WUR not only filters interference but also reduces the power consumption.

### 4.6. Comparison of Interference Mitigation Approaches

As shown in Figure 2 earlier, the interference mitigation schemes have been classified into five categories which are power control approach, MAC approach, cognitive radio approach, UWB approach, and signal processing approach. In this subsection, the five approaches are compared with each other in terms of robustness to mobility, lossy channel support, self-learning ability, effectiveness, and cost as shown in Table 4. The robustness to mobility is considered as the ability of a mitigation scheme in mobile environments and densely populated WBASNs. The lossy channel support is examined with respect to high fading channels. Effectiveness can be evaluated whether the technique is robust to the interference. Cost is evaluated on the basis of channel parameters, convergence time, and acceptable performance.

**Table 4:** Comparison of the five interference mitigation approaches.

Interference Mitigation Approach	Robustness to Mobility	Lossy Channel Support	Self-Learning Ability	Effectiveness	Cost
Power control approach	Yes	No	Yes	Medium	High
MAC approach	Yes	No	Yes	High	High
Cognitive radio approach	Yes	Yes	Yes	High	Low
UWB approach	Yes	Yes	No	High	Medium
Signal processing approach	No	Yes	No	Medium	High

## 5. Open Issues and Challenges

### 5.1. System Throughput

System throughput, which is the most important factor to evaluate system performance, is related to data rate and packet delivery ratio. The higher the system throughput is, the more reliable communication will be in any scenario. Mobile and high-density WBASNs adversely affect bandwidth utilization. Because one application of WBASNs is healthcare service in which each sensor conveys vital signals from a human body to the service center, signal loss may result in a dangerous situation for patients. Therefore, the first requirement of any mitigation scheme is to maximize system throughput.

### 5.2. Power Consumption

In order to ensure a long lifespan for the sensor nodes, energy efficiency or power consumption is one of the main goals in any WBASN application. The power capacities of sensor nodes are limited due to the small size of their batteries, which cannot be replaced or recharged. Moreover, in interference scenarios, the power consumption of a WBASN increases because of contention to access the channel, retransmission, and the idle listening channel. Some algorithms consider the minimization power in a WBASN as a main objective. However, a good tradeoff between performance and power consumption is needed to ensure that the interference mitigation schemes work effectively.

### 5.3. QoS and Reliability

WBASNs have specific QoS requirements for each type of sensor or application [1,7]. Specifically, the QoS constraint relies on bit error rate (BER) or the priority of a transmitted signal. In a scenario with interference in a hospital or a long queue in a public place, WBASNs with a high QoS constraint should have a high priority to access the channel because they may carry vital data about a chronic illness such as a heart disease.

Reliability is considered in terms of packet delay and probability of packet loss. The end-to-end delay of the received data depends on the WBASN application; for example, the delays for an ECG signal and a video signal must be below 250 ms and 20 ms, respectively [7]. Furthermore, in the case of interference among WBASNs, the convergence time in which WBASNs return to normal operation or stable status affects packet delay. The shorter the convergence time, the more effective the interference mitigation scheme; therefore, WBASN quickly returns to the stable operation leading to low end-to-end delay. In addition, the probability of packet loss determines the extent to which the packet drop rate affects the reliability of a WBASN in terms of BER or packet error rate.

### 5.4. Dynamic Environment

The mitigation scheme must work in a dynamic and mobile environment. WBASNs have the ability to suppress interference by acting independently without negotiating or exchanging their information with other WBASNs. In the case of negotiation methods, exchanging information between WBASNs may cause a long delay or slow convergence, but WBASNs perform better if they are aware of the whole network system. In the case of nonnegotiation methods, a WBASN needs to optimize its performance, converge quickly, and learn about the network system by itself.

### 5.5. Impact of Wireless Communication on the Human Body

Because the biosensors are placed in or on the human body, the radio frequency (RF) radiation could affect adversely to the human body. The RF radiation in interaction with biological systems has been investigated in terms of thermal and non-thermal bioeffect [50]. Average specific absorption rate (SAR) limitation in the whole human body is 0.4 W/kg and 0.08 W/kg in controlled and uncontrolled environments, respectively; local SAR is 1.6 W/kg in any gram tissue in the shape of a cube [51]. The thermal mechanisms result in temperature rise of the biological system; e.g., a continuous wave RF field of 30 V/m at 1 GHz produces SAR of about 1 W/kg [50]. One of the non-thermal mechanisms is membrane excitation; *i.e*., a very short pulse can cause membrane breakdown. It is conducted in [52] that the non-thermal mechanisms are not fully understood but they do not lead to the biological effects. Therefore, the human body may be concerned with the safety problem not only for a short term but also for a long term.

More specifically, in [53], the impact of RF radiation on the human body has been identified as well as classified according to the radiation sources, impacts, and factors. The radiation impacts are grouped into the extremely low frequency and radio frequency groups. Furthermore, the factors that affect RF absorption are categorized into physical parameters, biological parameters, artifacts, and environmental parameters. Consequently, some open research areas for passive wireless technologies have been addressed for healthcare environments. Another research has been conducted in three-dimensional wireless sensor networks in [54], in which the aspect of radiation awareness is investigated in three-dimensional environments. The main focus is to find the minimum radiation path for a person moving from one point to another point.

In WBASNs, the coordinator node is considered as a body-placed device which can transmit signals to the router. In [55], the new antenna structure which can reduce SAR is used in a body-worn communication device, and it can be also applied to the coordinator node. The newly designed antenna which is one type of an artificial magnetic conductor (AMC) has the slotted periodic structure. In addition, this type of antennas is used at a wideband code division multiple access as well. In [56], another type of AMC is used as an AMC-intergrated antenna which is designed for WBASNs to operate in the ISM bands. The simulation results in [55,56] show that the AMC antenna is suitable to reduce SAR on the human body.

However, it is necessary to find a path with low SAR in WBASNs, which is conducted in [57] by using an optimization algorithm. One node will be chosen to be the relay node which transmits signals from other sensor nodes to a hub by using the low SAR path.

## 6. Conclusions

In this survey, we have reviewed interference mitigation schemes for WBASNs. Coexistence of WBASNs has been revisited with respect to problems involving interference, and the existing inter-WBASN interference mitigation schemes have been categorized and qualitatively compared. Although many interference mitigation schemes have been reported, there is no dominating scheme that significantly outperforms the others. An analytical tradeoff between network throughput and power consumption in WBASNs is needed in the dynamic channels in WBASNs. Channel dynamics, QoS, delay, reliability, and network lifespan also must be considered with the design in terms of interference. Because the performance and QoS of WBASNs are severely affected by interference, various interference mitigation algorithms will be developed in the future. The cross-layer design of interference mitigation will be one direction. It should be noticed that the QoS requirement has not been fully investigated, and performance in some biomedical applications would be compromised with existing interference mitigation techniques.

## References

[1] Astrin A. (2012). IEEE Standard for Local and Metropolitan Area Networks Part 15.6: Wireless Body Area Networks.

[2] Tobón D.P., Falk T.H., Maier M. (2013). Context Awareness in WBANs: A Survey on Medical and Non-Medical Applications. IEEE Wirel. Commun..

[3] Mehfuz S., Urooj S., Sinha S. (2015). Wireless BODY Area Networks: A Review with Intelligent Sensor Network-Based Emerging Technology. Information Systems Design and Intelligent Applications.

[4] Movassaghi S., Abolhasan M., Lipman J., Smith D., Jamalipour A. (2014). Wireless Body Area Networks: A Survey. IEEE Commun. Surv. Tutor..

[5] (2006). IEEE Std. 802.15.4–2006. IEEE Standard for Information Technology—Local and Metropolitan Area Networks—Specific Requirements—Part 15.4: Wireless Medium Access Control (MAC) and Physical Layer (PHY) Specifications for Low Rate Wireless Personal Area Networks (WPANs).

[6] Lay-Ekuakille A., Griffo G., Vergallo P., Massaro A., Spano F., Gigli G. (2015). Implantable Neurorecording Sensing System: Wireless Transmission of Measurements. IEEE Sens. J..

[7] Cavallari R., Martelli F., Rosini R., Buratti C., Verdone R. (2014). A Survey on Wireless Body Area Networks: Technologies and Design Challenges. IEEE Commun. Surv. Tutor..

[8] De Silva B., Natarajan A., Motani M. Inter-User Interference in Body Sensor Networks: Preliminary Investigation and an Infrastructure-based Solution. Proceeding of the Sixth International Workshop on Wearable and Implantable Body Sensor Networks.

[9] Xuan W., Lin C. Interference Analysis of Co-existing Wireless Body Area Networks. Proceeding of the Global Telecommunications Conference (GLOBECOM 2011).

[10] Zhang A., Smith D.B., Miniutti D., Hanlen L.W., Rodda D., Gilbert B. (2010). Performance of Piconet Co-Existence Schemes in Wireless Body Area Networks. Proceeding of the Wireless Communications and Networking Conference (WCNC).

[11] Deylami M., Jovanov E. Performance Analysis of Coexisting IEEE 802.15.4-Based Health Monitoring WBANs. Proceeding of IEEE Conference on Engineering in Medicine and Biology Society (EMBC).

[12] Mucchi L., Carpini A. Aggregate Interference in ISM Band: WBANs Need Cognitivity?. Proceeding of the International Conference on Cognitive Radio Oriented Wireless Networks and Communications (CROWNCOM).

[13] Januszkiewicz L. Simplified Human Body Models for Interference Analysis in the Cognitive Radio for Medical Body Area Networks. Proceeding of the International Symposium on Medical Information and Communication Technology (ISMICT).

[14] Jin Z., Han Y., Cho J., Lee B. (2015). A Prediction Algorithm for Coexistence Problem in Multiple-WBAN Environment. Int. J. Distrib. Sens. Netw..

[15] Hernandez M., Miura R. Coexistence of IEEE Std 802.15.6TM-2012 UWB-PHY with Other UWB Systems. Proceeding of the IEEE International Conference on Ultra-Wideband (ICUWB).

[16] Martelli F., Verdone R. Coexistence Issues for Wireless Body Area Networks at 2.45 GHz. Proceeding of the 18th European Wireless Conference on European Wireless.

[17] Gengfa F., Dutkiewicz E., Kegen Y., Vesilo R., Yiwei Y. Distributed Inter-Network Interference Coordination for Wireless Body Area Networks. Proceeding of the 2010 IEEE Conference on Global Telecommunications Conference (GLOBECOM 2010).

[18] Kazemi R., Vesilo R., Dutkiewicz E., Gengfa F. Inter-Network Interference Mitigation in Wireless Body Area Networks using Power Control Games. Proceeding of the International Symposium on Communications and Information Technologies (ISCIT).

[19] Kazemi R., Vesilo R., Dutkiewicz E., Liu R.P. Reinforcement Learning in Power Control Games for Internetwork Interference Mitigation in Wireless Body Area Networks. Proceeding of the 2012 International Symposium on Communications and Information Technologies (ISCIT).

[20] Kazemi R., Vesilo R., Dutkiewicz E. A Novel Genetic-Fuzzy Power Controller with Feedback for Interference Mitigation in Wireless Body Area Networks. Proceeding of the 2011 IEEE Conference on Vehicular Technology.

[21] Zhang Z., Wang H., Wang C., Fang H. (2013). Interference Mitigation for Cyber-Physical Wireless Body Area Network System Using Social Networks. IEEE Trans. Emerg. Top. Comput..

[22] Chen Q., Su C., Zhang H., Chai R. User Service Oriented Power Allocation Algorithm for Wireless Body Area Sensor Networks. Proceeding of the 5th IET International Conference on Wireless, Mobile and Multimedia Networks (ICWMMN 2013).

[23] Zou L., Liu B., Chen C., Chen C.W. Bayesian Game Based Power Control Scheme for Inter-WBAN Interference Mitigation. Proceeding of the IEEE Global Communications Conference (GLOBECOM).

[24] Spanakis E.G., Sakkalis V., Marias K., Traganitis A. (2014). Cross Layer Interference Management in Wireless Biomedical Networks. Entropy.

[25] Deylami M.N., Jovanov E. (2013). A Distributed Scheme to Manage the Dynamic Coexistence of IEEE 802.15.4-Based Health-Monitoring WBANs. IEEE J. Biomed. Health Inform..

[26] Mahapatro J., Misra S., Manjunatha M., Islam N. Interference-Aware Channel Switching for Use in WBAN with Human-Sensor Interface. Proceeding of the 4th International Conference on Intelligent Human Computer Interaction (IHCI).

[27] Liang S., Ge Y., Jiang S., Tan H.P. A Lightweight and Robust Interference Mitigation Scheme for Wireless Body Sensor Networks in Realistic Environments. Proceeding of the IEEE Wireless Communications and Networking Conference.

[28] Movassaghi S., Abolhasan M., Smith D. Smart Spectrum Allocation for Interference Mitigation in Wireless Body Area Networks. Proceeding of the IEEE International Conference on Communications (ICC).

[29] Jamthe A., Mishra A., Agrawal D.P. Scheduling Schemes for Interference Suppression in Healthcare Sensor Networks. Proceeding of the IEEE International Conference on Communications (ICC).

[30] Kim E.J., Youm S., Shon T., Kang C.H. (2013). Asynchronous Inter-Network Interference Avoidance for Wireless Body Area Networks. J. Supercomput..

[31] Hwang H., Kim N. An Enhanced Frame Transmission Method for Health Devices with Ultra Low Power Operation. Proceeding of the IEEE International Conference on Consumer Electronics (ICCE).

[32] Cheng S.H., Huang C.Y. (2013). Coloring-Based Inter-WBAN Scheduling for Mobile Wireless Body Area Networks. IEEE Trans. Parallel Distrib. Syst..

[33] Chen G.T., Chen W.T., Shen S.H. 2L-MAC: A MAC Protocol with Two-Layer Interference Mitigation in Wireless Body Area Networks for Medical Applications. Proceeding of the IEEE International Conference on Communications (ICC).

[34] Yang W.B., Sayrafian K. Interference Mitigation for Body Area Networks. Proceeding of the 22nd International Symposium on Personal Indoor and Mobile Radio Communications (PIMRC).

[35] Xie Z., Huang G., He J., Zhang Y. (2014). A Clique-Based WBAN Scheduling for Mobile Wireless Body Area Networks. Proced. Comput. Sci..

[36] Movassaghi S., Abolhasan M., Smith D. Cooperative Scheduling with Graph Coloring for Interference Mitigation in Wireless Body Area Networks. Proceeding of the IEEE Wireless Communications and Networking Conference.

[37] Movassaghi S., Abolhasan M., Smithy D., Jamalipour A. AIM: Adaptive Internetwork Interference Mitigation Amongst Co-Existing Wireless Body Area Networks. Proceeding of IEEE Global Communications Conference (GLOBECOM).

[38] Shen Q., Liu J., Yu H., Ma Z., Li M., Shen Z., Chen C. Adaptive Cognitive Enhanced Platform for WBAN. Proceeding of the IEEE/CIC International Conference on Communications in China (ICCC).

[39] Han J., Liu J., Yu H., Chen C., Shen Z. HCVP: A Hybrid Cognitive Validation Platform for WBAN. Proceeding of the International Conference on Wireless Communications & Signal. Processing (WCSP).

[40] Nhan N.Q., Gautier M., Berder O. Asynchronous MAC Protocol for Spectrum Agility in Wireless Body Area Sensor Networks. Proceeding of the International Conference on Cognitive Radio Oriented Wireless Networks and Communications (CROWNCOM).

[41] Ouattara D., Quach M.T., Krief F., Chalouf M.A., Khalife H. Mitigating the Hospital Area Communication’s Interference using Cognitive Radio Networks. Proceeding of the IEEE 15th International conference on E-Health Networking, Applications & Services (Healthcom).

[42] Santiago R.C., Balasingham I. Cognitive Radio for Medical Wireless Body Area Networks. Proceeding of the 16th International Workshop on Computer Aided Modeling and Design of Communication Links and Networks (CAMAD).

[43] Syed A.R., Yau K.L.A. On Cognitive Radio-based Wireless Body Area Networks for Medical Applications. Proceedings of the IEEE Symposium on Computational Intelligence in Healthcare and E-Health (CICARE).

[44] Bae J.N., Choi Y.H., Kim J.Y., Kwon J.W., Kim D.I. (2011). Efficient Interference Cancellation Scheme for Wireless Body Area Network. J. Commun. Netw..

[45] Rout D.K., Das S. Multiple Narrowband Interference Mitigation in UWB Body Area Networks for Body Surface Communications. Proceeding of the International Conference on Medical Imaging, M-Health and Emerging Communication Systems.

[46] Labate D., Foresta F.L., Occhiuto G., Morabito F.C., Lay-Ekuakille A., Vergallo P. (2013). Empirical Mode Decomposition *vs.* Wavelet Decomposition for the Extraction of Respiratory Signal from Single-Channel ECG: A Comparison. IEEE Sens. J..

[47] Lay-Ekuakille A., Vergallo P., Trabacca A., de Rinaldis M., Angelillo F., Conversano F., Casciaro S. (2013). Low-Frequency Detection in ECG Signals and Joint EEG-Ergospirometric Measurements for Precautionary Diagnosis. Measurement.

[48] Ghanem K., Hall P.S. Interference Cancellation Using CDMA Multi-user Detectors for on-Body Channels, Personal. Proceeding of the IEEE 20th International Symposium on Indoor and Mobile Radio Communications.

[49] Marinkovic S.J., Popovici E.M. (2011). Nano-Power Wireless Wake-Up Receiver with Serial Peripheral Interface. IEEE J. Sel. Areas Commun..

[50] Foster K.R. (2000). Thermal and Nonthermal Mechanisms of Interaction of Radio-Frequency Energy with Biological Systems. IEEE Trans. Plasma Sci..

[51] (2002). IEEE Std. C95.3–2002. IEEE Recommended Practice for Measurements and Computations of Radio Frequency Electromagnetic Fields with respect to Human Exposure to such Fields, 100 kHz-300 GHz.

[52] Challis L.J. (2005). Mechanisms for Interaction between RF Fields and Biological Tissue. Bioelectromagnetics.

[53] Arumugam D.D., Gautham A., Narayanaswamy G., Engels D.W. Impacts of RF Radiation on the Human Body in a Passive Wireless Healthcare Environment. Proceeding of the 2nd International Conference on Pervasive Computing Technologies for Healthcare.

[54] Nikoletseas S., Patroumpa D., Prasanna V.K., Raptopoulos C., Rolim J. Radiation awareness in Three-Dimensional Wireless Sensor Networks. Proceeding of the IEEE 8th International Conference on Distributed Computing in Sensor Systems (DCOSS).

[55] Kwak S., Kwon J.H., Sim D.U., Choi H.D. Design of Improved Antenna with the Slotted Periodic Structures for SAR Reduction in Body-Worn Communication Device. Proceeding of the 2011 Asia-Pacific Microwave Conference Proceedings (APMC).

[56] Di Y.H., Liu X.Y., Tentzeris M.M. A Conformable Dual-band Antenna Equipped with AMC for WBAN Applications. Proceeding of the 3rd Asia-Pacific Conference on Antennas and Propagation (APCAP).

[57] Wu T.Y., Lin C.H. (2015). Low-SAR Path Discovery by Particle Swarm Optimization Algorithm in Wireless Body Area Networks. IEEE Sens. J..

